# Top 10 metrics for life science software good practices

**DOI:** 10.12688/f1000research.9206.1

**Published:** 2016-08-16

**Authors:** Haydee Artaza, Neil Chue Hong, Manuel Corpas, Angel Corpuz, Rob Hooft, Rafael C. Jimenez, Brane Leskošek, Brett G. Olivier, Jan Stourac, Radka Svobodová Vařeková, Thomas Van Parys, Daniel Vaughan

**Affiliations:** 1 The Earlham Institute & ELIXIR-UK, Norwich Research Park, Norwich, NR4 7UH, UK; 2 Software Sustainability Institute, University of Edinburgh, Edinburgh, EH9 3FD, UK; 3 University “La Sapienza, Rome, 00185, Italy; 4 DTL, PO Box 19245, Utrecht, 3501 DE, Netherlands; 5 ELIXIR Hub, Wellcome Trust Genome Campus, Hinxton, CB10 1SD, UK; 6 Institute for Biostatistics and Medical Informatics (IBMI), Faculty of Medicine, University of Ljubljana, Ljubljana, SI-1104, Slovenia; 7 Systems Bioinformatics, Vrije Universiteit Amsterdam, Amsterdam, 1081 HV, Netherlands; 8 Loschmidt Laboratories, Faculty of Science, Masaryk University, Brno, 625 00, Czech Republic; 9 CEITEC Masaryk University, Brno, 625 00, Czech Republic; 10 Department of Plant Systems Biology, VIB, Ghent, 9052, Belgium; 11 EMBL-EBI, Wellcome Trust Genome Campus, Hinxton, CB10 1SD, UK

**Keywords:** Software, Best Practices, Metrics, Evaluation, Impact, ELIXIR, Sustainability

## Abstract

Metrics for assessing adoption of good development practices are a useful way to ensure that software is sustainable, reusable and functional. Sustainability means that the software used today will be available - and continue to be improved and supported - in the future.

We report here an initial set of metrics that measure good practices in software development. This initiative differs from previously developed efforts in being a community-driven grassroots approach where experts from different organisations propose good software practices that have reasonable potential to be adopted by the communities they represent. We not only focus our efforts on understanding and prioritising good practices, we assess their feasibility for implementation and publish them here.

## Introduction

Compliance with and promotion of good development practice is a powerful mechanism for promoting software sustainability. Using metrics to judge good practice can enhance research software maintainability and helps establish a baseline of quality, reusability and reproducibility. Software development metrics, however, are only useful if it is clear what they measure. This could be a) the application of agreed good practice in a piece of software or software team, or b) how sustainable the software is in the long term. There have been previous attempts to assess good practices for scientific computing ^1^ but they did not specifically tackle the question how to measure them during software development. As part of a collaboration between the ELIXIR pan-European research infrastructure for life science data and the Software Sustainability Institute, a working group met at Schiphol airport (Amsterdam) on December 14–15th 2015 to a) define and select the metrics that reflect the **application** of good practices, b) discuss the collection of these metrics and c) establish how the metrics could be implemented to ensure their wide adoption. In this article we report the outcomes of this workshop. We believe this effort is set apart from previous initiatives because of its ‘bottom up’ approach to ensure community adoption and therefore it should have realistic chances of implementation. We benefit from the fact that participating members of both groups have long established track records in life science research software development. This is the first release of our agreed software development good practices and expect that new revisions could evolve from it in future iterations. It is outside of the scope of this manuscript to delve into the issues that these metrics might raise in terms of performance comparison between different software. 

## Methods

In a workshop 12 experts from across Europe met to discuss good software development practices for life sciences. At the meeting, the group was divided randomly into two equally large subgroups to facilitate discussion, each subgroup spending a set time discussing potential metrics, their impact and applicability. The experts in each subgroup did not impose any restriction on which metrics to propose, but rather aimed to be as inclusive as possible, as long as each suggested metric had potential for impact. After the discussion, each group summarised the results and subsequently we merged the resulting metrics together into a list of 17 topics.

Next, the two groups worked on prioritising the identified metrics according to two criteria: 1) Importance and 2) Implementability. *Importance* is a measure of the impact that a particular practice can have in making software more sustainable. A metric is considered highly *implementable* if it is easy to generate. For each identified metric, importance and implementability were ranked by all members of the working group on a scale from 1 to 5, 5 being highest importance or easiest implementation. An average score was calculated and the resulting list was sorted from highest to lowest scoring metrics. Here we discuss and evaluate a final list of the top ten prioritised metrics.

## Results

We identified a set of 17 topics that are critical for software development good practice ( Box 1). It was evident that these include measurements of different styles: measurements can be self-reported, automatically produced or externally audited. The type of metric is also important to consider here: there are metrics of qualitative and quantitative nature. Qualitative metrics correspond to a binary classification description, while quantitative metrics tend to be more amenable to integration and presentation as statistics. Metrics interpretation may pose challenges of its own kind, particularly related to the subjective nature of the importance of metrics and the different perceptions of value according to the context in which they are used.

Box 1. **Our complete list of potential topics to be indicative of good practices in software development.**

Each of these topics have quantitative and qualitative metrics that may help track the adoption of good practice and monitor compliance with the guidelines in life sciences.

1. **Version control:**
  a. Yes/no?
  b. How many committers?
  c. When was the version control started?
  d. When was the last commit?
2. **Code reviews:**
  a. Yes/no?
  b. Star rating based on code description
3. **Automated testing:**
  a. Yes/no?
  b. Coverage for unit tests
  c. Yes/no for individual tests:
    i. Unit tests
    ii. Functional tests
    iii. Integration tests
    iv. Regression tests
  d. Are the tests part of the code in the repository?
4. **Not reinventing the wheel:**
  a. Using libraries?
  b. Using Frameworks?
  c. Describing the algorithm, explaining why known code is reimplemented.
  d. Reinventing should be documented. References to the algorithm?
  e. Percentage of code written from scratch?
  f. Percentage of code that is involved in the core functionality?
5. **Discoverability:**
  a. Via structured search on functionality?
  b. Is it in the ELIXIR Tools and Data Services Registry ^2^ or others (e.g., BioSharing ^3^)?
6. **Reusability of source code:**
  a. Number or reuses = number of derived projects/external commits?
7. **Reusability of software:**
  a. Number of citations on the paper
  b. Having basic description of features in structured ELIXIR format (EDAM ontology ^4^) - in ELIXIR Tools and Data Services Registry?
8. **Licensing:**
  a. Is there a license?
  b. Is the source available?
  c. Is it open source according to opensource.org?
9. **Issue tracking/bug tracking:**
  a. Does it have a publicly accessible issue tracker?
  b. How long are issues open?
  c. What is the number of unresolved issues?
  d. How much activity has there been in the last three months in the issue tracker?
10. **Support processes:**
  a. Are basic processes defined? Like governance, mailing list, releases, ...
11. **Compliance with community standards:**
  a. Yes/no?
  b. Specifies the level of compliance, specification version or metrics?
12. **Buildable code:**
  a. Does the compiler give warnings?
  b. Does a static analysis (“lint”) give warnings?
  c. Is an automated build system used?
13. **Open development:**
  a. Number of external committers in the repositories
14. **Making data available:**
  a. Yes/no?
  b. Where?
15. **Documentation:**
  a. Ratio code/comments, code lines/document lines?
  b. Percentage of code dedicated to documentation?
16. **Simplicity:**
  a. Measure of cyclomatic complexity
17. **Dependency management:**
  a. Is it done automatically using a system?
  b. Does it use a language-standard repository to pull in dependencies?
  c. Is software made available as a dependency in a dependency repository?

We used the 43 metrics contained in the 17 identified topics as a basis for further prioritisation as described in the Methods section. Prioritisation of metrics was achieved by all participants scoring them according to their perception of importance and implementability. An average score was calculated and a sum of average importance and average implementability to rank the list ( Table 1). We introduced also a manual evaluation for each of the proposed ranked metrics, which reflected the consensus of the final prioritisation, given initial difference of opinions when reviewing the average scores. In Table 1, we summarise the top 10 suggested metrics.

**Table 1.** Prioritised top 10 metrics for assessment of life science software development good practice. Each identified metric was scored according to importance (for sustainability) and implementability. Importance scores ranged from 1 (little) to 5 (very much) and implementability from 1 (difficult) to 5 (easy). Average values are shown for both importance ( ^**a**^) and implementability ( ^**b**^). A priority score ( ^**c**^) is calculated as the sum of the averages provided by ( ^**a**^) and ( ^**b**^). ( ^**c**^) is further discussed and the final Manual Priority Evaluation ( ^**d**^) is agreed, reflecting the final prioritisation judgement decided by the Working Group.

Top 10 Ranked Metrics	Avg Importance ^a^	Avg Implementability ^b^	Avg Sum Priority Score ^c^	Manual Priority Evaluation ^d^
**Is version control used?**	5	4.6	9.6	1
**Is the software discoverable?**	4.1	5	9.1	2
**Is an automated build system used?**	4.6	3.9	8.4	3
**Are test data available?**	3.8	4	7.8	4
**Does software contain parts that** **reimplement existing technology?**	4.4	2.9	7.3	5
**Is the software compliant with** **community standards?**	4.1	2.5	6.6	6
**Are code reviews performed?**	3.4	2.8	6.1	7
**Is automated testing performed?**	3.5	3.1	6.6	8
**Is the code documented?**	2.4	4.3	6.6	9
**How high is the code complexity?**	3.5	2.9	6.4	10

As a use case, we base the application of these metrics within the context of code development in ELIXIR. We define each of the 10 prioritised metrics in Table 1 and, where necessary, describe and explain the motivation for a metric and how to measure it. We consider that these definitions are applicable to a wider range of software development communities in life sciences.

1. Is controlled versioning used?

○ **Description**: Is it clearly indicated, can it be easily found?
○ **Motivation**: Version control systems provide an environment for safe and transparent software development.
○ **How to measure**: Put information about a version control tool to the ELIXIR Tools and Data Services Registry (which system, when it was installed, …)

2. Is the software discoverable?

○ **Description**: Is it easy to find the software based on its functionality (without knowing its exact name)?
○ **Motivation**: It is important to be discoverable so other potential contributions are encouraged and more people use the software.
○ **How to measure**: The ELIXIR community should be motivated and guided to provide this information into the ELIXIR Tools and Data Services Registry. If not, a list of other catalogues should be defined (maximum 5–10 other sources, e.g. BioSharing, field-specific catalogues, etc.). If the tool cannot be found there, the discoverability should be evaluated as 0.

3. Is an automated build system used?

○ **Description**: Are the builds of the software performed by some automated system?
○ **Motivation**: If the automated system for builds is applied, can the users rebuild the software easily, which markedly increases its usability?
○ **How to measure**: This information should be again included into the ELIXIR Tools and Data Services Registry ^2^. Ideally, a link to the installation document should also be provided. How many commands are necessary for building of the software? (Optimally, just one command should be performed.)

4. Are test data available?

○ **Description**: Are data for testing of the software easily available for users?
○ **Motivation**: Without test data, it may be difficult to try the functionality of the software and assess correct functioning of an installation.
○ **How to measure**: The test data should be linked to from the web page describing the software or in the supplementary material of its associated publication. A link to the data should be included in ELIXIR Service Registry.

5. Does software contain parts that reimplement existing technology?

○ **Description**: Are common components/algorithms covered by libraries or reimplemented?
○ **Motivation**: A (naïve) reimplementation can cause unnecessary errors or decrease the effectiveness.
○ **How to measure**: Percentage of code written from scratch and/or number of used libraries. Additionally, descriptions of why a library with similar functionality was not used and responses to suggestions from community.

6. Does the software support open community standards and what is its level of compliance?

○ **Description**: Evaluation of software compliance with open/community standards
○ **Motivation**: This is needed, for example, where data input/output, networking and general interoperability are concerned. However, it is also non-trivial to implement and measure in terms of the overall software quality.
○ **How to measure**: A base metric would be: “does the software make use of open standards (yes/no), if so which ones (listing)?” In addition, more qualitative information such as “which versions of the standard does the software support?”, “Is it compatible with the latest specification?”, and “Can it be used to provide a more general level of support?” Another fundamental aspect to consider is whether the standard provide its own compliance metric (e.g., a test suite) and what the software’s level of compliance is. An example of such a compliance test suite is provided by the Systems Biology Markup Language (SBML, ^5^).

7. Are code reviews performed?

○ **Description**: Whether new code is inspected by someone else before it becomes part of the code base.
○ **Motivation**: Code reviews increase quality of the code both because it is written with more care and because the second pair of eyes will more readily catch false assumptions or errors.
○ **How to measure**: Activity in code review process (comments to updated lines, etc.)

8. Is automated testing performed?

○ **Description**: Is some system for automated testing implemented?
○ **Motivation**: Automatic testing decreases occurrence of bugs.
○ **How to measure**: Information about the testing methodology should be present in the software documentation. In parallel, developers can be motivated to add this information to ELIXIR Service Registry.

9. Is the code documented?

○ **Description**: Does the code contain comments describing its main elements?
○ **Motivation**: Code comments increase the readability of the code and also indirectly motivate the programmer to write a cleaner code. However, commenting can present the problem of not being updated as code changes. This means that code comments may rot and become misleading/inaccurate. Often comments can be made redundant by better names of variables and methods. An exception is example code where explaining what each line does with a comment is useful.
○ **How to measure**: Determine the percentage of text from the source code that corresponds to comments.

10. How high is the code complexity?

○ **Description**: This refers to how complex or straightforward the code is.
○ **Motivation**: The more complex code, the higher risk of errors. Code can be simplified by proper separation of tasks into different routines and methods.
○ **How to measure**: Measure the cyclomatic complexity.

## Discussion and conclusion

We present an initial set of 10 good practices that could help make software for the life sciences more sustainable. From our discussions, it was clear that a community-wide adoption of standards is needed in terms of how measurement of metrics are collected and shared. We operate under the assumption that all software developed should be open source from the beginning of development, which means that the collection of statistics for good practice compliance should not violate any of the licensing or privacy issues associated to closed code.

These ‘Top 10 Good Practices’ should be considered as an initial view of what the community considers important with a description of their feasibility for implementation within the life sciences. Among our top suggested topics there is a remarkable coincidence on the need for versioning. The ways on how to collect metrics regarding versioning systems vary: if using GitHub, a number of statistics are readily available that allow their easy collection for benchmarking. We do not, however, want to prescribe which versioning systems should be adopted. There are many ways in which this metric can be measured, a sample of which we offer. The metrics we propose can be both qualitative and quantitative. Although quantitative metrics are easier to track, it is also important to capture qualitative characteristics such as existence of automated testing or compliance with community standards.

This article is a first attempt to crystallise the conclusions from the work that the group of experts gathered under the auspices of ELIXIR and the Software Sustainability Institute. It is thus not intended to be a final declaration of what the ELIXIR community thinks the metrics, implementation and feasibility for measuring good practices for software development should be. This document is an initial response from the working group established to assess the problem of evaluating metrics for software development good practices. We expect it to be modified in future versions as more experts join this group and new challenges emerge with evolving technologies and life science software needs.
