# Supplementary material for: Top 10 metrics for life science software good practices
Source: F1000Res. 2016 Aug 16;5:ELIXIR-2000. [Version 1] doi: 10.12688/f1000research.9206.1 (PMC5007752; doi:10.12688/f1000research.9206.1)
Supplement: Peer review history

# Peer review: Referee response for version 1

**DOI:** 10.5256/f1000research.9909.r15704

**Published:** 2016-08-26

**Authors:** Bruno Gaëta

**Affiliations:** 1 School of Computer Science and Engineering, University of New South Wales, Sydney, NSW, Australia

As a meeting report and opinion piece, there is little to contradict in the manuscript. The ideas put forward are sensible and make a great starting point for discussion which is the purpose of the manuscript. Maybe the scope of the article could be slightly better defined to differentiate the goal of the proposed metrics from that of the metrics and methods used for software verification (see for example Giannoulatou et al (2014) )

I have read this submission. I believe that I have an appropriate level of expertise to confirm that it is of an acceptable scientific standard.

## References

[1] GiannoulatouEParkSHHumphreysDTHoJW: Verification and validation of bioinformatics software without a gold standard: a case study of BWA and Bowtie. *BMC Bioinformatics*.2014;15 Suppl 16: 10.1186/1471-2105-15-S16-S15 S15 10.1186/1471-2105-15-S16-S15 25521810PMC4290646

# Peer review: Referee response for version 1

**DOI:** 10.5256/f1000research.9909.r15707

**Published:** 2016-08-24

**Authors:** Pedro Fernandes

**Affiliations:** 1 Instituto Gulbenkian de Ciência, Oeiras, Portugal

This is a pragmatic report that describes a systematic approach to selecting criteria to measure the adoption of good practices in software development. The context of this study is a not as wide as the title and abstract indicate. However, the assessments are fair and the methodology for collecting data is well described. The use case provides a ground for a concrete reflection, and that is very useful. The generalisation of the method will require more decoupling from the use case, but it is understood that it can happen in future iterations of this study.

 It is reasonable to believe that  various grades of agreement has been reached in the discussions.  It would have been interesting to have included some measurement of dispersion, as the prioritisation results from ranking average scores alone (Table 1). It would add value to the confidence in the choice of the top 10 prioritised metrics that otherwise look indistinguishable from a full consensus. Although it is useful to concentrate on the top 10 and describe them at this level of detail, it would be very useful to see the ranking of the full set of 43 metrics analysed, possibly in a bar graph with the average score and error bars to indicate dispersion. That would enrich this manuscript significantly.

 The importance of this effort in defining such criteria is very large, and, as the authors suggest, it represents a first step of an iterative process that is much needed in this area.  In subsequent iterations some refinement of the measurement methods will be needed, such as in metric #9. "Is the code documented?", presently listed as "How to measure: Determine the percentage of text from the source code that corresponds to comments."  The advantage of having the metric in usage is high, but the way to measure needs to match the relative importance of the criteria. In this case, a high percentage of text in comments is relevant is the comments are useful and contextualised, and it can be argued that a large quantity of irrelevant comments can actually be detrimental in various aspects, from code readability to maintainability itself.

I have read this submission. I believe that I have an appropriate level of expertise to confirm that it is of an acceptable scientific standard.
